# Efficacy of pulsed-xenon ultraviolet light for disinfection of high-touch surfaces in an Ecuadorian hospital

**DOI:** 10.1186/s12879-019-4200-3

**Published:** 2019-07-03

**Authors:** José E. Villacís, Mario Lopez, Deborah Passey, Manuel Hernando Santillán, Germán Verdezoto, Freddy Trujillo, Gustavo Paredes, Carmen Alarcón, Ronny Horvath, Mark Stibich

**Affiliations:** 1 Science department, 360Life Technologies, 6 de Diciembre y Batallas, Quito, Ecuador; 2 Carrera de Bioquímica Clínica, Facultad de Medicina, Pontificia Universidad Católica del Ecuador, Quito, Ecuador; 3 Hospital Enrique Garcés, Quito, Ecuador; 4 Xenex Disinfection Services, San Antonio, Texas 78209 USA

**Keywords:** UV-C light, HAIs, Disinfection, Environmental surfaces, Carbapenemase

## Abstract

**Background:**

Hospital environment in patient care has been linked on healthcare-associated infections (HAI). No touch disinfection technologies that utilize pulsed xenon ultraviolet light has been recognized to prevent infection in contaminated environments. The purpose of this study was: 1) to evaluate the effectiveness of pulsed-xenon ultraviolet light (PX-UV) disinfection for the reduction of bacteria on environmental surfaces of Hospital General Enrique Garcés, and 2) to evaluate the in-vitro efficacy against multi-drug resistance microorganisms.

**Methods:**

This was a quality-improvement study looking at cleaning and disinfection of patient areas. During the study, a total of 146 surfaces from 17 rooms were sampled in a secondary 329-bed public medical center. Microbiological samples of high-touch surfaces were taken after terminal manual cleaning and after pulsed xenon ultraviolet disinfection. Cleaning staff were blinded to the study purpose and told clean following their usual protocols. For positive cultures PCR identification for carbapenemase-resistance genes (*blaKPC, blaIMP, blaVIM*, and *blaNDM*) were analyzed and confirmed by sequencing. The total number of colony forming units (CFU) were obtained and statistical analyses were conducted using Wilcoxon Rank Sum tests to evaluate the difference in CFU between terminal manual cleaning and after pulsed xenon ultraviolet disinfection.

**Results:**

After manual disinfection of 124 surfaces showed a total of 3569 CFU which dropped to 889 CFU in 80 surfaces after pulsed xenon disinfection (*p* < 0.001). Overall, the surface and environmental contamination was reduced by 75% after PX-UV compared to manual cleaning and disinfection. There were statistically significant decreases in CFU counts of high touch surfaces in OR 87% (*p* < 0.001) and patient rooms 76% (*p* < 0.001). Four rooms presented serine carbapenemases *blaKPC*, and metallo beta-lactamases *blaNDM, blaVIM, blaIMP*. confirmed by PCR and sequencing. The in-vitro testing with endemic strains found that after five minutes of pulsed xenon ultraviolet exposure an 8-log reduction was achieved in all cases.

**Conclusion:**

This study is one of the first of its kind in an Ecuador Hospital. We found that pulsed-xenon ultraviolet disinfection technology is an efficacious complement to the established manual cleaning protocols and guidelines in the significant reduction of MDRO.

## Background

Healthcare-associated infections (HAIs) are an important public health threat due to the high rate of morbidity and mortality [1]. In Latin America countries, HAIs caused by microorganisms where the treatment options are limited can significantly prolong the hospital length of stay and increase healthcare costs [2, 3]. The fight against multidrug-resistant microorganisms (MDROs) offers only short-term solutions due to the high capacity for resistance and dissemination [4]. However, new prevention strategies may provide a more effective solution for combating infections caused by MDROs.

The role of healthcare workers in the transmission of microorganisms from patient to patient is well documented, however, there is growing evidence that high-touch surfaces are a significant source for transmission of pathogens that lead to HAIs. [5, 6] Consistent cleaning and disinfection of high-touch surfaces may prevent HAIs. In order to maintain effectiv**e**ness, manual cleaning and disinfection procedures require constant education and supervision of the environmental service (EVS) personnel [7]. Chemical disinfectants with or without detergents are a standard way to improve surface hygiene in clinical and hospital areas, but there is variation in the way EVS staff use and apply chemical disinfectants. For example, EVS staff may not be trained on the types of dilutions required for different surfaces or on the order of application to avoid cross-contamination. Additionally, EVS staff may have time constraints that do not allow for chemicals to have the proper dwell time on surfaces. The use of chemical disinfectants also presents occupational health risks for EVS staff that use them [8]. Due to these limitations of chemical disinfectants for manual cleaning, no-touch systems for disinfection and environmental decontamination are being considered the standard of care. One type of no-touch disinfection system uses pulsed-xenon ultraviolet light (PX-UV) to generate germicidal wavelengths of light (200-280 nm), which has shown to be 95 to 99% effective in eliminating hospital pathogens, including MDROs, from high touch surfaces and has been associated with significant reductions in HAIs [9–16].

The objective of the present study was to 1) evaluate the effectiveness of the PX-UV system, in the reduction of bacteria on environmental surfaces of Hospital Enrique Garcés after routine terminal manual cleaning and disinfection and after PX-UV disinfection, and 2) assess the in-vitro efficacy against multi-drug resistant microo**r**ganisms.

## Methods

This quality-improvement study for disinfecting environmental surfaces was conducted at the Enrique Garcés General Hospital in Quito, Ecuador. The hospital is a second-level public hospital with 329 beds and 33 specialties (clinics, surgical, gynecological, obstetric) that provides ambulatory, in-patient, and emergency services for the diagnosis, treatment, and recovery of patients. We received approval for the study from the hospital’s Institutional Review Board (IRB) and was conducted in an empty patient room, and no patient’s data was involved in any way.

Onsite visits included meetings with the care management team, heads of service, microbiology laboratory, and nursing. In addition, 17 hospital rooms were sampled: 4 OR (21 surfaces), 8 rooms ICU (57 surfaces), 2 rooms Internal medicine (10 surfaces), Neo-ICU (34 surfaces), Neo-Infectology (12 surfaces) and microbiology lab (2 samples).

Once the rooms were identified, communication channels were created with chief nurses to notify staff for sampling and disinfection after patient discharge. Hospital cleaners performed terminal manual cleaning and disinfection using a 2500 ppm (0.25%) chlorine disinfectant for 20 min according to the hospital protocol. Cleaning staff were blinded to prevent any changes in cleaning practices.

Microbiological samples were taken using trypticase soy agar (TSA) contact plates with 5 mm diameter (Hardy Diagnostics, P-34, Santa Maria, CA). We followed the manufacturer’s instructions for sampling surfaces, for flat surfaces pressure method was used and for curved surfaces the rolling plate technique was used to ensure sampling of the entire area. Microbiological samples were taken after terminal manual cleaning and after pulsed xenon ultraviolet disinfection on adjacent surfaces.

The PX-UV disinfection was deployed for one five-minute cycle in the bathroom, two five-minute cycles in individual patient rooms, and two 10-min cycles in operating rooms. The contact plates were incubated for 48 ± 4 h at 35 ± 2 °C. The counts of individual colonies were made after 24 and 48 h of incubation under photographic record. For positive cultures, the strains recovered by surface were recorded by room location and PCR identification for carbapenemase resistance genes *bla*KPC, *bla*IMP, *bla*VIM, and *bla*NDM were explored using primers described elsewhere [17] and confirmed by sequencing.

Frequencies and the total number of colony forming units (CFU) were obtained after terminal manual cleaning and after PXUV-C disinfection, in aggregate and by surface location. Each colony, regardless of color or morphology was recorded for heterotrophic mesophilic bacteria counts. We made comparisons for all surfaces per operating and patient rooms and statistical analyses were conducted using Wilcoxon Rank Sum tests using RStudio V1.1.463 (RStudio Inc.). *p*-values less than .05 were considered statistically significant.

For the in-vitro study, endemic strains to the hospital were used; *S. aureus* (*MRSA*), *E. faecium* (*Van B*), *Pseudomonas aeruginosa* (*VIM*), and *Klebsiella pneumoniae* (*KPC*) were selected. After having a 24-h pure colony, dilutions were made in saline solution of 10^8^, 10^6^, 10^4^ and 10^2^ for subsequent inoculation, swabbing the surface of the agar completely with a standardized concentration of CFU according the Kirby Bauer method in Mueller Hinton agar in duplicate. The petri dishes with and without lids were exposed to PX-UV disinfection at one meter for one five-minute cycle. Petri dishes were then incubated at 35 ± 2 °C with the results read after 24 h.

## Results

**Fig. 1:**
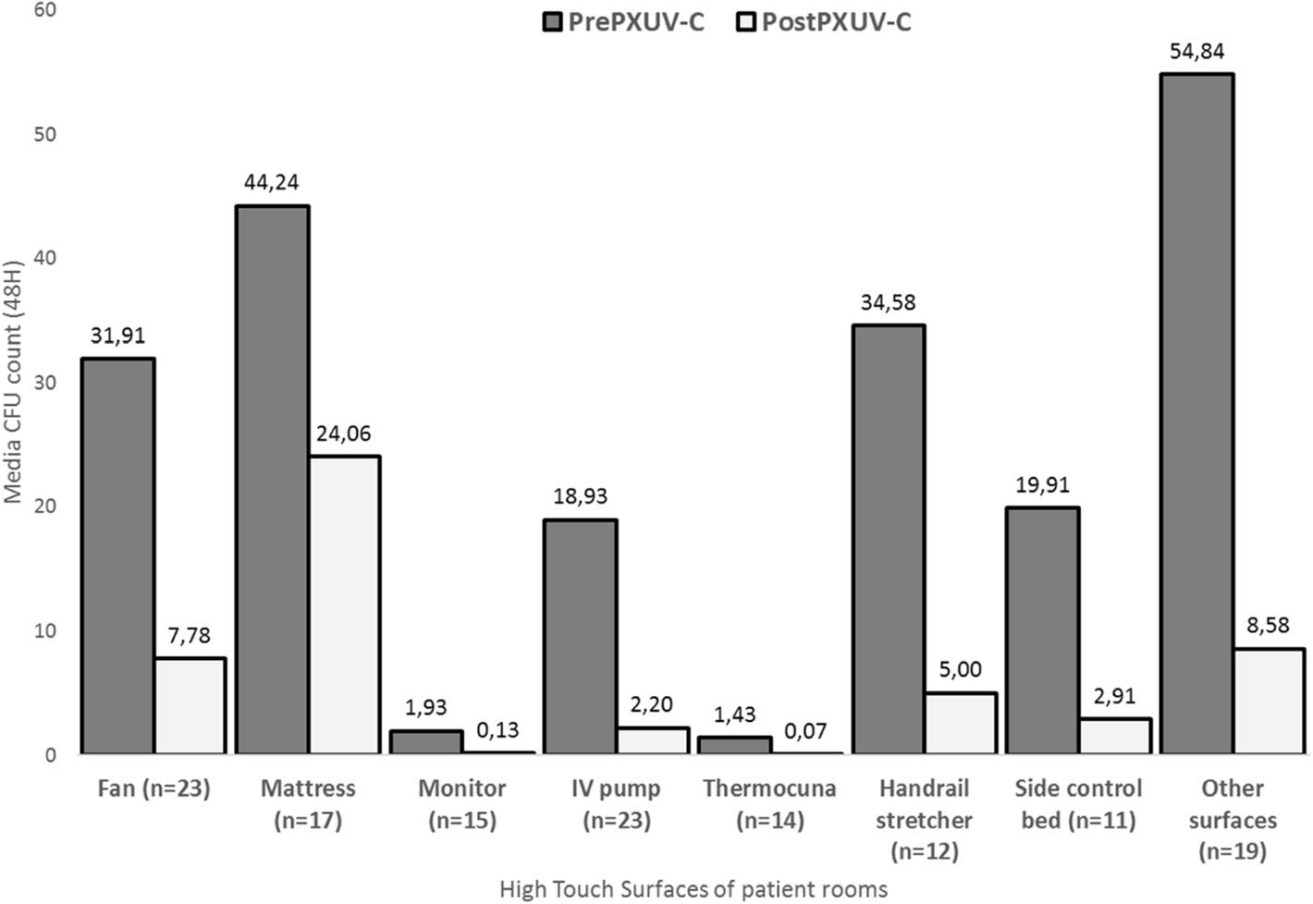
Average in CFU of High Touch Surfaces of patient rooms. On the horizontal axis are the surfaces sampled, and the vertical axis is expressed average of Colony counts units (CFU). °Other surfaces: external air duct, workbench, curtain, sink, door handle, soap dispenser, bath rail, bathroom sink, shower faucet, bathroom switch, bedside table, table

**Fig. 2:**
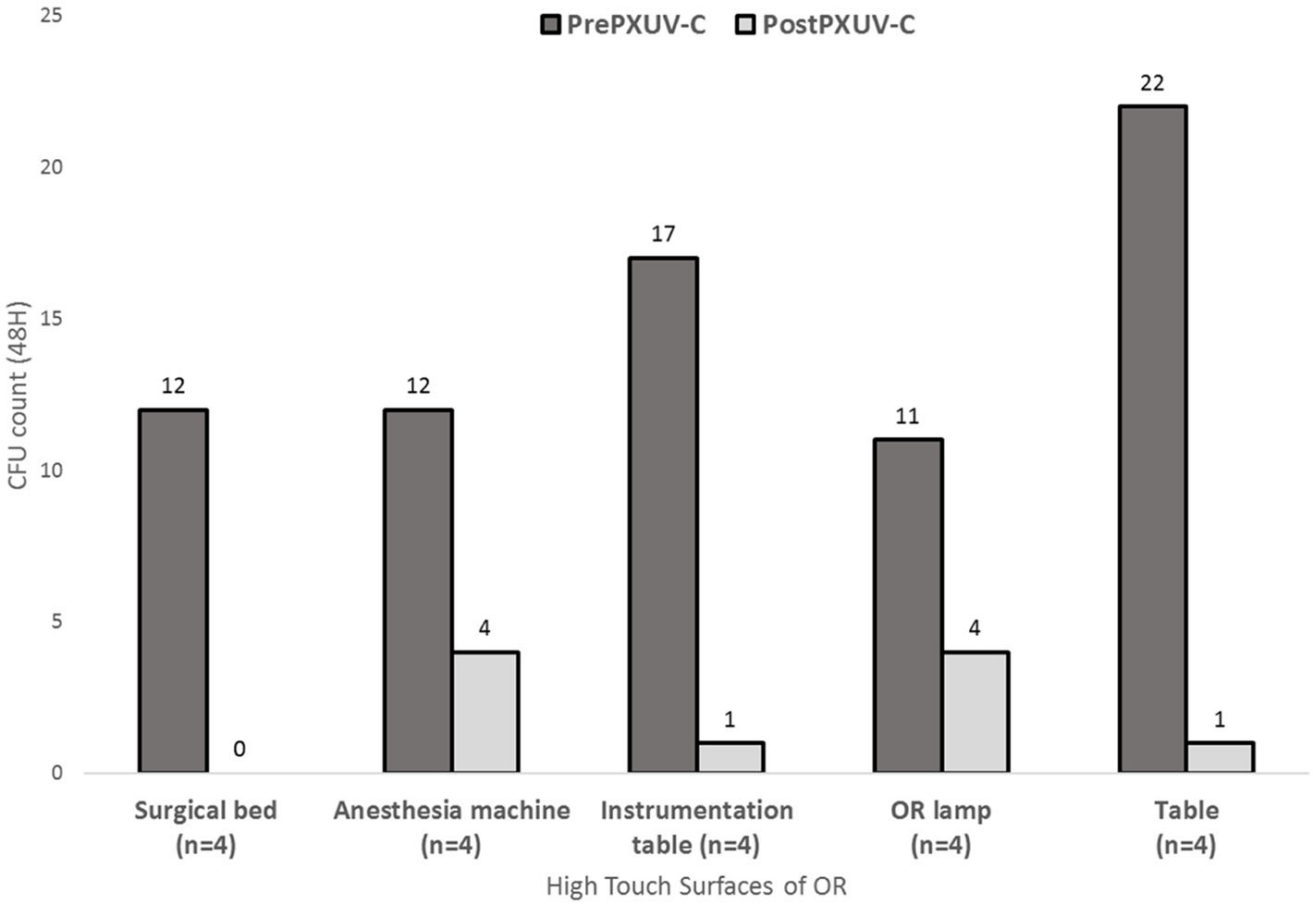
Total CFU on High Touch Surfaces of OR. On the horizontal axis are the surfaces sampled, and the vertical axis is expressed number of Colony counts units (CFU)

A total of 146 surfaces from 17 rooms were sampled after manual cleaning and disinfection, and after PX-UV disinfection (internal medicine, ICU, operating rooms, neonatology, obstetric center, and microbiology laboratory units). After manual disinfection 124 surfaces showed a total of 3569 CFU, which dropped to 889 CFU in 80 surfaces after PX-UV disinfection. Overall, the surface and environmental contamination was reduced by 75% (*p* < 0.001) after PX-UV compared to manual cleaning and disinfection. There was statistically significant reduction of CFU counts on operating rooms 87% (*p* < 0.001) and patient rooms 76% (*p* < 0.001). (Table 1, Fig. 1, Fig. 2).

**Table 1:** Summary statistics for OR Room and Patient Rooms (ICU, Internal Medicine and Neonatology) from all surfaces sampled

Service (*n* = 16)		Total CFU	Mean ± SD	Median (Min-Max)
OR Room (*n* = 4)	After manual disinfection	76	3.61 ± 2.67	3 (0–8)
	After PX-UV	11	0.52 ± 0.93	0 (0–3)
	Percent reduction	87%		
	Wilcoxon signed rank	164		
	*p*-value	< 0.001		
Patient Room (*n* = 12)	After manual disinfection	3488	28.36 ± 56.54	10 (0–487)
	After PX-UV	877	7.13 ± 22.73	1 (0–230)
	Percent reduction	76%		
	Wilcoxon signed rank	5135		
	*p*-value	< 0.001		

*PX-UV* pulsed-xenon ultraviolet light, *CFU* colony form units

Before PX-UV disinfection, 17 rooms contaminated with aerobic bacteria were screened for carbapenamase resistance genes. According to the PCR amplification, four rooms presented: serine carbapenemase *blaKPC*, and metallo beta-lactamases *blaNDM*, *blaVIM*, *blaIMP*_._ confirmed by PCR and sequencing (Table 2). The data obtained for in-vitro testing with MDROs with the presence of resistance genes showed a high bactericidal efficacy in all four strains. After five minutes of PX-UV exposure an 8-log reduction was achieved in all cases (Table 3).

**Table 2:** Carbapenemase detection in different locations of Hospital

Service	Location	Carbapenemase	Allele
Internal Medicine	MI702	metallo-β-lactamase	IMP-15, NDM-1
Neonatology	Neo UCI	metallo-β-lactamase	IMP-5, VIM-53
		serine carbapenemase	KPC-2
	Neo Infectology	metallo-β-lactamase	VIM-2
Internal Medicine	MI706	metallo-β-lactamase	IMP-15, VIM-2

**Table 3:** Laboratory testing results

Microorganism	Resistance Mechanism	Pre PX-UV	Post PX-UV	Log Reduction
*Klebsiella pneumoniae*	*Klebsiella pneumoniae* carbapenemase (KPC)	1.5E+ 08	1.50E+ 01	1.49 E+ 08 99%
*Pseudomonas aeruginosa*	Verona Integron-Mediated Metallo-beta-lactamase (VIM)	1.5E+ 08	1.00E+ 00	1.49 E+ 08 99%
*Staphylococcus aureus*	mecA gene	1.5E+ 08	8.00E+ 00	1.49 E+ 08 99%
*Enterococcus faecium*	vanB gene	1.5E+ 08	2.00E+ 00	1.49 E+ 08 99%

## Discussion

It is now recognized that the environment bioburden plays a significant role in the transmission of HAIs. Overall, our results provide strong evidence that the hospital environment is a major reservoir that contributes to the risk of HAIs. The presence of bacteria in different high touch surfaces in OR and patient rooms is similar with other studies that show that bacteria can persist in hospital locations and equipment [12, 13, 18]. In this study, we report the presence of carbapenemase resistance genes *blaKPC*, *blaNDM*, *blaVIM*, *blaIMP* in the environment of internal medicine and neonatology before PX-UV disinfection. This contaminated high touch surfaces may contribute to the transmission of MDROs with carbapenemases which have been previously reported in clinical cases in Ecuadorian hospitals [4, 19, 20].

Our findings suggest that the use of PX-UV disinfection significantly reduced environmental contamination by 75% beyond manual cleaning alone. The high percentage of reduction of microbial bioburden propose that no-touch disinfection technologies are an important adjunct to the manual cleaning process of hospital environment [9, 12, 15, 16, 21]. The surfaces properly disinfected with an adequate hand hygiene compliance is recommended to prevent the transfer of pathogens to healthcare workers hands that has been shown to play a significant role in the acquisition of HAIs [22].

The in-vitro study carried out with MDROs also showed that one 5-min cycle is sufficient to eliminate bacteria under laboratory conditions. These data are important for implementing no-touch disinfection technologies to enhance terminal manual cleaning and disinfection after every patient discharge.

Our findings are important for the development of appropriate disinfection protocols in hospital settings using PX-UV to prevent HAIs. However, there is no published research on the implementation of no-touch disinfection technology in a Latin American healthcare system. To our knowledge, this is the first study in Ecuador to evaluate the effectiveness of the PX-UV system in the reduction of bacteria present on environmental surfaces from a Hospital of Quito-Ecuador.

Limitations of the study include that was conducted at a single hospital site, and the rooms sampled may not be representative of other hospitals in Ecuador. We only collected samples from a small sample of operating rooms (*n* = 4) and patient rooms (*n* = 12). This study was not designed to assess the impact of hospital-acquired infections. Our study had strengths, including the variety of MDROs tested in-vitro and the results obtained are similar to bioburden decrease seen in hospitals studies of U. S and United Kingdom.

## Conclusion

This study provides evidence that the addition of pulsed xenon UV-C light is an efficacious complement to the established manual cleaning protocols and guidelines of hospitals. We believe that the addition of pulsed xenon UV-C light to the disinfection protocols of the hospitals, can reduce enormously the human errors that the traditional method entails. The deployment of pulsed PX-UV devices is feasible in Ecuador and Latin America countries based on these results which demonstrate both the inadequacy of manual cleaning and the ability of the technology to improve the environment. The speed at which this technology is adopted will depend on effective surveillance systems for HAIs and incentives, such as those launched in the United States to improve the quality of patient care. Future studies are needed to evaluate the economic, clinical, and institutional impact of PX-UV on prevention and control of HAIs.

## Data Availability

The data analyzed for this paper will be available from corresponding author on reasonable request.

## Abbreviations

CFU: Colony forming units
HAIs: Hospital Acquire Infections
MDRO: Multidrug resistant organisms
OR: Operating room
PXUV: Pulsed Xenon Ultraviolet Light

## References

[1] World Health Organization (WHO). Global burden of infections associated with health care. WHO; 2013. [cited 2018 Oct 6]; Available from: https://www.who.int/gpsc/country_work/burden_hcai/es/

[2] Grundmann H, Aires-de-Sousa M, Boyce J, Tiemersma E (2006). Emergence and resurgence of meticillin-resistant *Staphylococcus aureus* as a public-health threat. Lancet.

[3] Allegranzi B, Nejad SB, Combescure C, Graafmans W, Attar H, Donaldson L (2011). Burden of endemic health-care-associated infection in developing countries: systematic review and meta-analysis. Lancet.

[4] Escandón-Vargas K, Reyes S, Gutiérrez S, Villegas MV (2017). The epidemiology of carbapenemases in Latin America and the Caribbean. Expert Rev Anti-Infect Ther.

[5] Dancer SJ (2009). The role of environmental cleaning in the control of hospital-acquired infection. J Hosp Infect.

[6] Weber DJ, Rutala WA (2013). Understanding and preventing transmission of healthcare-associated pathogens due to the contaminated hospital environment. Infect Control Hosp Epidemiol.

[7] Carling PC, Parry MF, Von Beheren SM, Group HEHS (2008). Identifying Opportunities to Enhance Environmental Cleaning in 23 Acute Care Hospitals. Infect Control Hosp Epidemiol.

[8] Dettenkofer M, Spencer RC (2007). Importance of environmental decontamination--a critical view. J Hosp Infect.

[9] Stibich M, Stachowiak J, Tanner B, Berkheiser M, Moore L, Raad I (2011). Evaluation of a pulsed-xenon ultraviolet room disinfection device for impact on hospital operations and microbial reduction. Infect Control Hosp Epidemiol.

[10] Nerandzic MM, Thota P, Sankar CT, Jencson A, Cadnum JL, Ray AJ (2015). Evaluation of a pulsed xenon ultraviolet disinfection system for reduction of healthcare-associated pathogens in hospital rooms. Infect Control Hosp Epidemiol.

[11] Haas JP, Menz J, Dusza S, Montecalvo MA (2014). Implementation and impact of ultraviolet environmental disinfection in an acute care setting. Am J Infect Control.

[12] Hosein I, Madeloso R, Nagaratnam W, Villamaria F, Stock E, Jinadatha C (2016). Evaluation of a pulsed xenon ultraviolet light device for isolation room disinfection in a United Kingdom hospital. Am J Infect Control.

[13] Green C, Pamplin JC, Chafin KN, Murray CK, Yun HC (2017). Pulsed-xenon ultraviolet light disinfection in a burn unit: impact on environmental bioburden, multidrug- resistant organism acquisition and healthcare associated infections. Burns.

[14] Jinadatha C, Quezada R, Huber TW, Williams JB, Zeber JE, Copeland LA (2014). Evaluation of a pulsed-xenon ultraviolet room disinfection device for impact on contamination levels of methicillin-resistant Staphylococcus aureus. BMC Infect Dis.

[15] Stibich BM (2016). Reduction of healthcare associated infections through the use of pulsed xenon ultraviolet disinfection.

[16] Levin J, Riley LS, Parrish C, English D, Ahn S (2013). The effect of portable pulsed xenón ultraviolet light after terminal cleaning on hospital-acquired Clostridium difficile infection in a community hospital. Am J Infect Control.

[17] Poirel L, Walsh TR, Cuvillier V, Nordmann P (2011). Multiplex PCR for detection of acquired carbapenemase genes. Diagn Microbiol Infect Dis.

[18] Simmons S, Dale C, Holt J, Passey DG, Stibich M (2018). Environmental effectiveness of pulsed-xenon light in the operating room. Am J Infect Control.

[19] Romero-Alvarez D, Reyes J, Quezada V, Satán C, Cevallos N, Barrera S, Trueba G, Escobar LE, Villacís JE (2017). First case of New Delhi metallo-β-lactamase in Klebsiella pneumoniae from Ecuador: An update for South America. Int J Infect Dis.

[20] Villacís JE, Bovera M, Romero-Alvarez D, Cornejo F, Albán V, Trueba G, Dorn HF, Reyes JA (2019). NDM-1 carbapenemase in Acinetobacter baumannii sequence type 32 in Ecuador. New Microbes New Infect.

[21] Simmons S, Morgan M, Hopkin T, Helsabeck K, Stachowiak J, Stibich M (2013). Impact of a multi-hospital intervention utilizing screening, hand hygiene education and pulsed xenon ultraviolet (PX-UV) on the rate of hospital associated meticillin resistant Staphylococcus aureus infection. J Infect Prev.

[22] Peters et al (2018). Keeping hospitals clean and safe without breaking the bank; summary of the healthcare cleaning forum 2018. Antimicrob Resist Infect Control.

